# Epstein–Barr Virus Detection in Lymphoproliferative Disorders: Epidemiological Characterization in Western Mexico

**DOI:** 10.3390/idr17040100

**Published:** 2025-08-14

**Authors:** Karel Cesar Licona-Lasteros, Eduardo Navarrete-Medina, Karina Franco-Topete, Sergio Yair Rodriguez-Preciado, Jaime Palomares-Marin, Gerardo Cazarez-Navarro, Ramón Antonio Franco-Topete, Iván Isidro Hernández-Cañaveral

**Affiliations:** 1 Programa de Doctorado en Microbiología Médica, Centro Universitario de Ciencias de la Salud, Universidad de Guadalajara, Guadalajara 44340, Jalisco, Mexico; karel.licona@alumnos.udg.mx; 2 Departamento de Microbiología y Patología, Centro Universitario de Ciencias de la Salud, Universidad de Guadalajara, Guadalajara 44340, Jalisco, Mexico; eduardo.navarrete5905@academicos.udg.mx (E.N.-M.); karina.topete@academicos.udg.mx (K.F.-T.); jaime.palomares@academicos.udg.mx (J.P.-M.); 3 Servicio de Anatomía Patológica, Hospital Civil de Guadalajara “Dr. Juan I. Menchaca”, Guadalajara 44340, Jalisco, Mexico; 4 Laboratorio de Sistemas Biológicos, Departamento de Ciencias de la Salud, Centro Universitario de los Valles, Universidad de Guadalajara, Ameca 46708, Jalisco, Mexico; sergio.rodriguezp@academicos.udg.mx; 5 Secretaria de Salud Jalisco, Dr. Baeza Alzaga 107, Zona Centro, Guadalajara 44100, Jalisco, Mexico; gecana55@hotmail.com

**Keywords:** Epstein–Barr virus, lymphoproliferative disorders, epidemiology, resource-limited settings, Mexico, molecular diagnostics

## Abstract

Background/Objectives: Epstein–Barr virus (EBV) detection patterns in lymphoproliferative disorders (LPDs) show significant geographical variation worldwide. Regional epidemiological data are essential for understanding viral distribution patterns and developing appropriate clinical surveillance strategies. This study aimed to determine EBV detection frequency in LPDs using available molecular and immunohistochemical methods in Western Mexico. Methods: We conducted a cross-sectional study of 200 formalin-fixed paraffin-embedded tissue samples from patients diagnosed with LPDs (2015–2019) at Hospital Civil de Guadalajara. EBV detection combined with real-time PCR targeting the BNTp143 gene and immunohistochemistry for LMP-1 protein. Cases were classified following current WHO criteria. Statistical analysis included multivariate logistic regression, diagnostic concordance assessment, and age-stratified analysis. Results: EBV detection frequency reached 35.5% overall, with marked differences between neoplastic (53.9%) and reactive LPDs (24.2%) (OR: 3.515; 95% CI: 1.859–6.645, *p* < 0.001). Hodgkin lymphoma showed the highest detection rate (80.6%), significantly exceeding non-Hodgkin lymphoma (39.3%) (OR: 6.43; 95% CI: 2.08–19.41, *p* = 0.001). Age-stratified analysis revealed predominant adult involvement (49.1% vs. 22.0% in young adults, *p* = 0.025). We identified three epidemiological categories based on detection probability patterns. Conclusions: This study represents the first comprehensive molecular and immunohistochemical characterization of Epstein–Barr virus in lymphoproliferative disorders from Western Mexico, establishing distinct epidemiological patterns that align with Latin American regional characteristics. The validated methodology provides a reproducible framework for multi-center studies, while the epidemiological data serve as an essential baseline for future longitudinal research and resource optimization in similar healthcare settings.

## 1. Introduction

Epstein–Barr virus (EBV), classified as human herpesvirus 4, establishes lifelong infection in more than 95% of the global adult population [1]. The epidemiological patterns of EBV detection in lymphoproliferative disorders (LPDs) demonstrate remarkable geographical and demographic variations that reflect complex interactions between viral, host, and environmental factors [2].

Geographic studies reveal striking regional differences in EBV detection rates in lymphomas. Latin American populations consistently show higher EBV detection rates (60–80%) in Hodgkin lymphoma compared to European (36%) or North American (32%) populations [3,4]. These patterns suggest regional-specific factors, including socioeconomic conditions, age of primary viral exposure, and potentially genetic predisposition [5]. In Mexico specifically, despite evidence of higher EBV seroprevalence among children compared to more affluent populations [6], comprehensive molecular and immunohistochemical characterization of EBV in LPDs remains limited.

The accurate detection of EBV in lymphoid tissues requires robust diagnostic approaches. While EBER in situ hybridization is widely considered a reference method, comparative studies have demonstrated that polymerase chain reaction (PCR) may achieve superior detection sensitivity. Qi et al. reported detection rates of 74.6% for PCR, 67.8% for EBER-ISH, and 66.1% for LMP-1 immunohistochemistry in Hodgkin lymphoma, establishing PCR as a highly sensitive detection method [7]. This finding supports the validity of PCR-based approaches for epidemiological surveillance, particularly in settings where EBER-ISH may not be routinely available.

Recent international consensus recommendations specifically validate the use of combined molecular and immunohistochemical approaches in regions with intermediate to high disease incidence [8]. The combination of molecular and immunohistochemical approaches provides complementary detection capabilities, with real-time PCR offering high sensitivity for viral DNA detection and LMP-1 immunohistochemistry providing cellular localization and expression pattern information.

The accurate diagnosis and classification of lymphoproliferative disorders present significant challenges in resource-limited environments. Studies from low- and middle-income countries report diagnostic error rates up to 40% when relying solely on routine hematoxylin and eosin staining without immunohistochemistry [9]. The prolonged turnaround times for pathological diagnosis (extending up to 10 weeks in many settings) further complicate clinical management [10]. These challenges underscore the importance of developing robust, resource-appropriate diagnostic strategies that can provide reliable epidemiological data while accommodating practical constraints [11].

EBV represents a particularly valuable diagnostic marker in lymphoproliferative disorders, especially in T/NK cell lymphomas, where conventional immunohistochemistry may be insufficient. For extranodal NK/T-cell lymphoma, nasal type (ENKTL), EBV in situ hybridization shows positivity in almost all cases, making it an essential diagnostic tool [12]. The broad spectrum of EBV-associated lymphoid neoplasms, including Burkitt lymphoma, angioimmunoblastic T-cell lymphoma, and various immunodeficiency-related lymphomas, emphasizes the clinical utility of comprehensive EBV detection approaches [11].

Mexico’s demographic and epidemiological characteristics suggest potentially unique EBV detection patterns. Studies from the United States have documented higher EBV prevalence rates among children of Mexican descent compared to other ethnic groups, particularly under conditions of lower socioeconomic status [13]. However, systematic characterization of EBV detection in Mexican patients with LPDs using validated molecular and immunohistochemical methods has not been comprehensively investigated.

The development of region-specific epidemiological data is crucial for several reasons. First, it enables appropriate clinical surveillance strategies tailored to local disease patterns. Second, it provides baseline data for monitoring temporal trends and therapeutic outcomes. Third, it informs resource allocation and diagnostic infrastructure development in healthcare systems [14].

Given these considerations, we conducted this comprehensive cross-sectional study with three primary objectives: (1) to determine the frequency of EBV detection in lymphoproliferative disorders in Western Mexico using validated molecular (real-time PCR) and immunohistochemical (LMP-1) methods; (2) to analyze demographic and histopathological distribution patterns of EBV-positive cases; (3) to characterize epidemiological patterns of EBV detection based on observed demographic and histopathological associations.

This study addresses significant knowledge gaps in regional EBV epidemiology while employing methodologically sound, resource-appropriate diagnostic approaches validated in international literature.

## 2. Materials and Methods

### 2.1. Study Design and Ethical Considerations

We conducted a cross-sectional study analyzing formalin-fixed paraffin-embedded (FFPE) tissue samples from patients diagnosed with lymphoproliferative disorders at the Anatomical Pathology Service of Hospital Civil de Guadalajara “Dr. Juan I. Menchaca,” a tertiary referral center serving Western Mexico. This study included archival material collected between January 2015 and December 2019.

This study was conducted in accordance with the Declaration of Helsinki and approved by the Hospital’s Research Ethics Committee (Ethics clearance number: 0146). The need for individual informed consent was waived following national research guidelines for studies using archived pathological specimens.

### 2.2. Histopathological Examination and Classification

Tissue sections were cut at 5 μm thickness and stained with hematoxylin and eosin. Two experienced pathologists with over 10 years of lymphoma diagnostics experience independently reviewed all cases following the current WHO classification of lymphoid neoplasms. Cases were systematically categorized as Hodgkin lymphoma, non-Hodgkin lymphoma, reactive lymphoid proliferations (including lymphoid hyperplasia and infectious associations), and other hematological neoplasms.

Reactive lymphoid proliferations were further classified into lymphoid hyperplasia and infectious association. “Infectious association” refers to reactive lymphadenopathies with specific histopathological evidence of identifiable infectious processes, characterized by distinctive morphological patterns that allow identification of causative agents or inflammatory response types. Unlike “lymphoid hyperplasia”, which represents non-specific responses without identifiable agents.

Sample selection criteria: Inclusion criteria included all lymphoproliferative disorders with adequate tissue for molecular analysis (available sections of at least 20 μm). Cases with insufficient tissue, poor-quality DNA (GAPDH-negative), or incomplete histopathological characterization were excluded. Diagnostic discrepancies between pathologists (observed in 5% of cases) were resolved by joint microscopic review and consensual discussion with a third pathologist, paying special attention to borderline reactive/neoplastic cases.

### 2.3. DNA Extraction Protocol

DNA extraction from FFPE tissue followed a standardized protocol using the xylene-ethanol method. We processed 20 μm tissue sections with 1 mL xylene treatment, followed by centrifugation at 13,000 rpm for 10 min and two washing cycles with 100% ethanol. DNA extraction was performed using the High Pure PCR Template Preparation Kit (Roche Diagnostics GmbH, Mannheim, Germany), with final DNA elution in 200 μL volumes and storage at −20 °C until analysis.

### 2.4. EBV Detection Methods

Following established protocols validated in the international literature [7,15], we employed two methods for comprehensive EBV detection.

Real-time PCR Analysis: We targeted the BNTp143 EBV gene using specific primers (Forward primer: 5′-GGAACCTGGTCATCCTTTGC-3′; Reverse primer: 5′-ACGTGCATGGACCGGTTAAT-3′), and TaqMan probe sequences (5′-/56-FAM/CGCAGGCACTCGTACTGCTCGCT/3BHQ_1/-3′). Based on robust scientific evidence and validated previous experience from our research team, BNTp143 was demonstrated as a highly effective target for EBV detection by real-time PCR, with a detection limit of ≤1 plasmid/10 μL and superior sensitivity compared to conventional PCR. In addition, there is a highly conserved sequence in the EBV genome with optimized 74 bp amplicon, ideal for FFPE tissues [16]. PCR conditions included an initial hold at 95 °C for 400 s, followed by 45 cycles of denaturation at 95 °C (10 s), annealing at 58 °C (15 s), and extension at 72 °C (25 s). The Human GAPD (GAPDH) Endogenous Control (VIC™/MGB probe) by Applied Biosystems ™ (Foster City, CA, USA) served as an internal positive control to verify DNA quality and amplification efficiency.

Quality Control Measures: Each PCR run included positive controls (EBV-positive cell line DNA), negative controls (water and EBV-negative tissue), and no-template controls. We used LightCycler^®^ Nano (Roche, North Ryde, Australia) with LightCycler^®^ TaqMan^®^ Master reagents (Roche, Cat No. 04 735 536 001). All samples were analyzed in duplicate, with results considered positive when both replicates showed amplification curves crossing the threshold before cycle 40. Inter-run reproducibility was verified using standard dilutions of positive control DNA.

Immunohistochemistry Protocol: We performed automated immunohistochemistry using Monoclonal Mouse Anti-Epstein–Barr Virus, LMP-1, clones CS.1-4 (combination of 4 anti-LMP-1 monoclonal antibodies. Isotype: IgG1, kappa chain) from Dako (Santa Clara, CA, USA), code no. M 0897. Positivity criterion: any specific cytoplasmic staining in morphologically appropriate cells; the presence of specific staining considered positive, consistent with international guidelines [12]; along with a comprehensive immunophenotyping panel, including CD1, CD3, CD5, CD10, CD15, CD20, CD30, CD34, CD45, CD68, CD138, BCL2, Ki67, and TdT. Immunostaining was performed using an automated immunostainer (Ventana BenchMark ULTRA, Ventana Medical Systems, Tucson, AZ, USA) with standard antigen retrieval and antibody dilutions according to manufacturer recommendations [17].

Definition of EBV positivity: Cases were considered EBV-positive if either PCR or IHC showed positive results. This combined approach was designed to maximize detection sensitivity while providing complementary biological information: PCR detection indicates the presence of the viral genome with high analytical sensitivity, IHC detection indicates active viral protein expression and cellular localization, and the combined interpretation captures viral infection status.

Recent international consensus recommendations for EBV-based screening explicitly validate the use of combined molecular and immunohistochemical approaches, particularly in regions with intermediate to high disease incidence [8].

### 2.5. Statistical Analysis

Statistical analysis was performed using IBM SPSS Statistics for Windows, version 26.0 (IBM Corp., Armonk, NY, USA). We employed descriptive statistics (frequencies and percentages for categorical variables; mean, standard deviation, median, and interquartile range for continuous variables) and inferential statistics, including Chi-square or Fisher’s exact test for categorical comparisons, independent *t*-test or ANOVA for continuous variables, and multivariate logistic regression incorporating LPD type, sex, and age groups. Statistical significance was set at *p* < 0.05, with 95% confidence intervals calculated for odds ratios.

## 3. Results

### 3.1. Patient Demographics and Clinical Characteristics

This study included 200 patients with lymphoproliferative disorders, demonstrating a slight female predominance (58.0%, *n* = 116). Mean age was 28.27 years (SD: 22.117), with a median of 25 years (IQR: 15–45) and age range 1–89 years. Age referenced corresponds to the age at initial histopathological diagnosis, with all samples processed within 48 h of diagnosis, ensuring no significant temporal difference. The most frequent anatomical location was cervical (62.0%, *n* = 124), followed by unspecified sites (13.5%, *n* = 27) and inguinal region (10.5%, *n* = 21) (Table 1).

### 3.2. Histopathological Distribution

**Figure 1:**
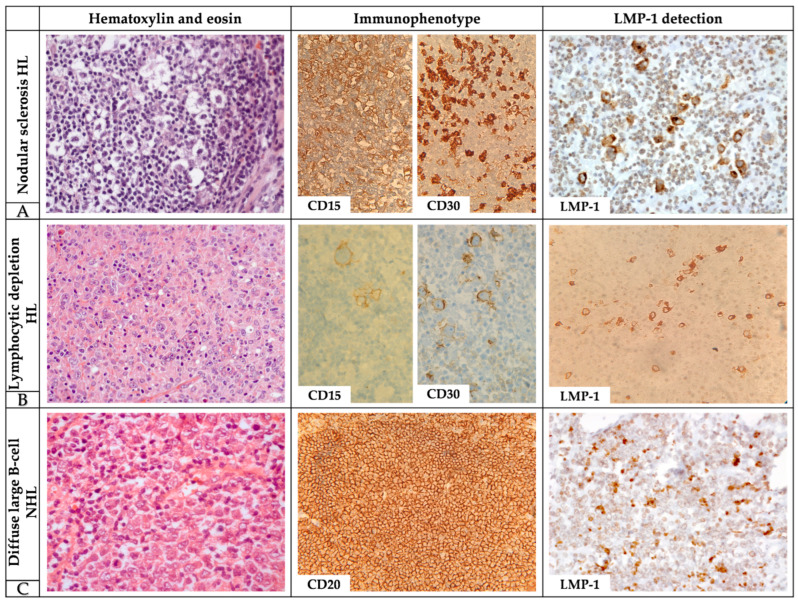
Representative morphology for LMP-1-positive lymphomas: (**A**) nodular sclerosis HL in HE (40×), CD15, CD30 (60×), and LMP-1 (40×); (**B**) lymphocytic depletion HL in HE (40×), CD15, CD 30 (40×), and LMP-1 (60×); (**C**) diffuse large B-cell NHL in HE (40×), CD20 (60×), and LMP-1 (60×). HL: Hodgkin Lymphoma; NHL: Non-Hodgkin Lymphoma; HE: Hematoxylin and Eosin; LMP-1: Latent Membrane Protein 1.

Histopathological review revealed reactive LPDs in 62.0% (*n* = 124) and neoplastic LPDs in 38.0% (*n* = 76). Specific diagnostic distribution included lymphoid hyperplasia (45.5%, *n* = 91), infectious association (16.5%, *n* = 33), Hodgkin lymphoma (15.5%, *n* = 31), non-Hodgkin lymphoma (14.0%, *n* = 28), and other neoplasms (8.5%, *n* = 17). Representative morphological features and immunohistochemical findings of LMP-1-positive lymphomas are shown in Figure 1.

### 3.3. EBV Detection Patterns

Overall EBV detection by either method reached 35.5% (71/200), with significantly higher rates in neoplastic (53.9%) versus reactive LPDs (24.2%) (adjusted OR: 3.515; 95% CI: 1.859–6.645, *p* < 0.001) (Table 2).

Hodgkin lymphoma demonstrated the highest detection rate (80.6%, 25/31), significantly exceeding non-Hodgkin lymphoma (39.3%, 11/28) (OR: 6.43; 95% CI: 2.08–19.41, *p* = 0.001). Within Hodgkin lymphoma subtypes, mixed cellularity showed 90.0% positivity (9/10), nodular sclerosis 80.0% (12/15), lymphocyte depletion 50.0% (2/4), and lymphocyte-rich 100.0% (2/2).

Non-Hodgkin lymphoma detection rates varied by subtype: diffuse large B-cell lymphoma 40.0% (4/10), follicular lymphoma 40.0% (2/5), T-cell lymphomas 50.0% (2/4), and Burkitt lymphoma 33.3% (1/3) (Table 3).

Reactive Conditions: Among reactive LPDs, infectious association processes showed higher EBV detection (39.4%, 13/33) compared to lymphoid hyperplasia (18.7%, 17/91), reflecting expected patterns in inflammatory conditions. Table 3 provides a comprehensive overview of the distribution of EBV positivity across these categories, highlighting the heterogeneity of EBV association in lymphoproliferative disorders.

### 3.4. Diagnostic Concordance Between Detection Methods

The PCR-BNTp143 assay demonstrated the highest sensitivity, detecting 61 out of 200 cases (30.5% overall positivity). In contrast, LMP-1 immunohistochemistry (IHC) exhibited a significantly lower detection rate, with only 23 positive cases out of 200 (11.5% overall positivity). When both methods were combined, the detection rate increased to 35.5% (71/200 cases), suggesting that a multimodal approach enhances diagnostic sensitivity (Table 4).

LMP-1 IHC showed perfect specificity (100%) relative to PCR as no cases were PCR-negative but IHC-positive (0/200). However, the sensitivity of IHC compared to PCR was limited, identifying only 23 out of 61 PCR-confirmed cases (37.7%).

Cohen’s kappa statistic was calculated to evaluate intermethod agreement, yielding a value of 0.42 (95% CI: 0.28–0.56, *p* = 0.004), which indicates moderate agreement between the two diagnostic techniques.

### 3.5. Age-Stratified Analysis

EBV detection demonstrated significant age-dependent patterns (*p* = 0.025): adults (31–60 years), 49.1% (27/55); elderly (>60 years), 40.0% (10/25); children (0–15 years), 34.4% (21/61); young adults (16–30 years), 22.0% (13/59).

Detailed stratification by LPD type revealed distinct patterns (Table 5). In reactive LPDs, adults showed significantly higher detection than young adults (40.0% vs. 7.7%; OR: 8.00; 95% CI: 2.134–27.81, *p* = 0.001), while children demonstrated intermediate detection rates (26.7%). In neoplastic LPDs, EBV distribution was more uniform across age groups, ranging from 45.0% in elderly patients to 65.0% in adults.

### 3.6. Epidemiological Patterns of EBV Detection

Based on identified demographic and histopathological associations, we characterized distinct epidemiological patterns of EBV detection in our regional population. High-probability detection pattern: Adults (31–60 years) with neoplastic lymphoproliferative disorders demonstrated the highest detection rates (65.0% in neoplastic vs. 40.0% in reactive LPDs). In reactive cases, this age group showed significantly elevated detection compared to younger adults (OR: 8.00; 95% CI: 2.134–27.81, *p* = 0.001). 

Intermediate-probability detection pattern: Young adults (16–30 years) across all histological classifications (22.0% overall), plus pediatric cases (<16 years) (34.4% overall), showing variable but consistent viral presence patterns. Children demonstrated higher detection rates than young adults but lower than middle-aged adults. Low-Probability detection pattern: EBV-negative cases across all age groups, representing the majority of reactive lymphoproliferative disorders (75.8%) and a substantial proportion of neoplastic cases (46.1%). Epidemiological utility: These patterns provide a framework for (1) optimizing diagnostic resource allocation in resource-limited settings; (2) prioritizing molecular testing based on demographic and histological factors; (3) establishing baseline data for longitudinal studies; and (4) regional epidemiological comparisons and international collaboration. Important limitation: This cross-sectional analysis provides epidemiological observations of viral presence patterns but cannot establish clinical risk predictions or therapeutic implications, which require prospective longitudinal validation.

**Table 5:** Clinical characteristics by LPD type and EBV positivity.

Characteristic	Reactive LPD				Neoplastic LPD			
	EBV(+) *n* (%)	EBV(−) *n* (%)	OR (95% CI)	*p*-Value	EBV(+) *n* (%)	EBV(−) *n* (%)	OR (95% CI)	*p*-Value
Sex								
Male	12 (21.8%)	43 (78.2%)	0.79 (0.35–1.75)	0.67	16 (55.2%)	13 (44.8%)	1.08 (0.44–2.73)	>0.99
Female	18 (26.1%)	51 (73.9%)	Reference		25 (53.2%)	22 (46.8%)	Reference	
Age Group								
Children	12 (26.7%)	33 (73.3%)	Reference	1.00	9 (56.2%)	7 (43.8%)	Reference	1.00
Young adults	3 (7.7%)	36 (92.3%)	0.22 (0.06–0.85)	0.04 *	10 (50.0%)	10 (50.0%)	0.77 (0.22–3.03)	0.74
Adults	14 (40.0%)	21 (60.0%)	1.83 (0.72–4.84)	0.23	13 (65.0%)	7 (35.0%)	1.44 (0.36–4.99)	0.73
Elderly	1 (20.0%)	4 (80.0%)	0.68 (0.05–5.08)	>0.9	9 (45.0%)	11 (55.0%)	0.63 (0.18–2.45)	0.73
Location								
Cervical	21 (29.2%)	51 (70.8%)	Reference	1.00	28 (53.8%)	24 (46.2%)	Reference	1.00
Inguinal	4 (25.0%)	12 (75.0%)	0.80 (0.26–2.71)	>0.9	2 (40.0%)	3 (60.0%)	0.57 (0.09–3.01)	0.65
Unspecified	3 (20.0%)	12 (80.0%)	0.60 (0.16–2.34)	0.54	8 (66.7%)	4 (33.3%)	1.71 (0.48–5.58)	0.52
Others	2 (9.5%)	19 (90.5%)	0.25 (0.05–1.09)	0.08	3 (42.9%)	4 (57.1%)	0.64 (0.15–2.61)	0.69

OR: Odds Ratio; CI: Confidence Interval. “Others” in the Location category includes axillary, mediastinal, and other less common sites. Statistical significance was set at * *p* < 0.05.

## 4. Discussion

Our findings demonstrate epidemiological patterns consistent with broader trends observed in developing regions worldwide. The overall detection frequency of 35.5% aligns with the elevated EBV detection rates characteristic of low- and middle-income countries, where infectious agents contribute to approximately 25% of cancer cases [2]. This pattern reflects the complex relationship between socioeconomic factors, early viral exposure, and subsequent malignancy development observed across similar epidemiological contexts [4].

The relationship between detection method sensitivity and clinical significance represents a critical consideration in EBV epidemiological studies. Qi et al. demonstrated PCR detection rates of 74.6% compared to 67.8% for EBER-ISH and 66.1% for LMP-1 immunohistochemistry in Hodgkin lymphoma [7]. The nature of PCR and immunohistochemistry addresses distinct diagnostic needs: PCR provides high sensitivity for viral genome detection, while immunohistochemistry offers valuable information about protein expression patterns and cellular localization. This approach is particularly relevant for resource-limited settings where optimal diagnostic infrastructure may not be consistently available, as emphasized in recent reviews of molecular oncology testing in such environments [11]. Recent work by Mangiaterra et al. demonstrated that highly sensitive methods can detect EBV transcripts (LMP1 and/or EBNA2) in cases previously considered EBV-negative by conventional approaches, identifying viral material in 29.17% of diffuse large B-cell lymphoma cases originally classified as EBV-negative [18]. However, their findings revealed important distinctions between trace viral presence and pathogenically relevant infection: cases with conventional EBER-ISH positivity showed significantly higher LMP1 protein expression and viral loads compared to those with only transcript detection [18].

This observation provides valuable context for interpreting our detection rates. While our combined PCR and immunohistochemical approach may detect viral presence across a spectrum of viral activity levels, the clinical and pathogenic relevance likely varies significantly. Our DLBCL detection rate of 40.0% substantially exceeds the 7% reported in other Mexican populations using EBER-ISH [19], suggesting that our more sensitive approach may be detecting cases with lower viral burdens that might not represent pathogenic involvement. This underscores the importance of interpreting our findings as epidemiological observations of viral presence rather than definitive evidence of viral pathogenesis.

Table 6 provides a systematic comparison of EBV detection rates across different geographical regions, demonstrating the distinct epidemiological profile of our Mexican population.

This comprehensive comparison reveals several important epidemiological patterns across global populations:

Sub-Saharan African studies demonstrate exceptionally high EBV detection rates, with Teshome et al. reporting near-universal detection (99%) in both Hodgkin and non-Hodgkin lymphomas using qPCR methodology [20]. This likely reflects endemic EBV-associated malignancies, where early viral exposure and cofactor interactions (including malaria and HIV co-infection) contribute to higher pathogenic involvement [21,22]. Zambian studies showed elevated but more moderate rates (40.9% HL, 54.5% NHL) using conventional PCR [22].

Latin American populations consistently show elevated detection rates (65–90% for Hodgkin lymphoma), with our Western Mexico findings (80.6%) aligning closely with regional reports from Central Mexico (71.4%), Brazil (65–75%), and Peru (90%). Asian studies demonstrate intermediate to high rates (67.8–74.6% for Hodgkin lymphoma), while European/North American populations consistently show lower detection rates (30–45% for Hodgkin lymphoma, 5–8% for DLBCL), reflecting distinct epidemiological profiles of developed regions.

Non-Hodgkin lymphoma patterns are more heterogeneous, reflecting subtype-specific associations, with notable differences between endemic African Burkitt (92–94%) and sporadic variants like ours (33.3%). Our combined real-time PCR and LMP-1 immunohistochemistry approach provides comprehensive detection capabilities while remaining feasible in resource-limited settings, addressing critical diagnostic needs where specialized techniques may not be consistently available [11,14].

The inclusion of African data provides crucial global context, with extremely high Ethiopian rates (99%) and elevated Zambian/Rwandan rates aligning with known Sub-Saharan African EBV-associated malignancy burden, emphasizing the importance of regional studies and supporting EBV-targeted therapeutic development in high-burden populations [20,21,22]. Our Mexican findings occupy an intermediate global position, with rates higher than European/North American but lower than some African studies, reflecting complex genetic, environmental, and socioeconomic factors influencing EBV pathogenesis. The methodological consistency across PCR-based studies supports the reliability of these epidemiological observations [7,20].

The age-stratified patterns observed in our study reflect complex interactions between host immune competence, viral exposure history, and malignancy development. The higher detection rates in adults (31–60 years) compared to young adults may reflect cumulative viral exposure effects, age-related immune changes, or cohort-specific exposure patterns. These findings align with similar age-related patterns reported in other Latin American populations [5].

The relatively high detection rates in children (34.4%) are consistent with the known higher EBV seroprevalence in Mexican pediatric populations compared to more affluent regions [6]. This pattern may reflect earlier primary infection and different viral–host interactions in our regional population.

The epidemiological patterns identified in our study provide a foundation for several important research directions. The development of circulating tumor DNA (ctDNA) and liquid biopsy approaches for lymphoma monitoring represents a promising frontier, particularly in resource-limited settings where repeated tissue sampling may be challenging [31]. Recent advances in molecular monitoring technologies offer potential applications for our regional context. EBV viral load quantification through real-time PCR can serve as a valuable biomarker for disease progression and future therapeutic research involving tabelecleucel and other targeted therapies. This study includes 25 cases of EBV-positive Hodgkin lymphoma (representing the primary indication) and 11 cases of EBV-positive non-Hodgkin lymphoma (constituting an exploratory cohort). These findings establish a critical epidemiological framework for designing clinical trials in analogous populations, highlighting the significant proportion of patients who may benefit from adoptive T-cell therapies.

Our findings underscore the importance of including populations from low- and middle-income countries in international lymphoma research initiatives. The lack of multicenter global reproducibility data for many diagnostic technologies emphasizes the need for collaborative efforts to validate tools and methods across diverse populations [9]. Such collaborations are essential for developing diagnostic approaches that are both scientifically robust and practically applicable across different healthcare environments.

Our study highlights the complex relationship between viral detection sensitivity and pathogenic significance. The enhanced sensitivity of our combined PCR and immunohistochemical approach likely contributes to higher detection rates compared to studies using EBER-ISH alone. This methodological difference is particularly relevant for interpreting our DLBCL findings, where our 40% detection rate substantially exceeds previous reports from similar populations.

Highly sensitive molecular methods can detect EBV material across viral activity spectrums, from pathogenically relevant infections to trace viral presence with unclear clinical significance [18]. The elevated detection rates observed may reflect both genuine regional epidemiological differences and methodological sensitivity variations [3], though the consistent pattern across multiple lymphoma subtypes suggests regional factors contribute significantly to these observations. However, the magnitude of difference in some categories (particularly DLBCL) indicates that methodological factors also play an important role [32]. While clinical decision-making should incorporate additional factors, including viral load levels and protein expression patterns [14], this represents the first comprehensive molecular and immunohistochemical characterization of EBV in lymphoproliferative disorders from Western Mexico, addressing a critical regional epidemiological knowledge gap.

Our study has important limitations that require acknowledgment. The cross-sectional design prevents causal inferences or clinical outcome predictions, representing associations at a single time point without informing disease progression or therapeutic response. Results from a single tertiary center serving Western Mexico limit generalizability, as geographic, genetic, and socioeconomic factors may influence viral–host interactions differently across regions. The absence of longitudinal follow-up prevents prognostic assessment, while our high-sensitivity combined PCR-IHC approach may detect viral presence across broad activity spectrums where clinical significance varies substantially. The substantial proportion of PCR+/IHC- cases (19.0%) requires careful interpretation, as distinguishing between latent infection and microenvironment presence needs additional methodological approaches. Population limitations include restrictions to HIV-negative cases per institutional protocols and potential hospital-based selection bias, while methodological heterogeneity complicates international comparisons with EBER-ISH or different PCR-based studies.

Our systematic approach establishes a robust foundation for future research directions, including longitudinal outcome studies correlating EBV status with clinical progression, therapeutic response analyses, expansion to other Mexican regions, and investigation of host genetic factors contributing to observed patterns. The standardized methodology ensures reproducibility in other centers and enables replication studies across different geographical regions, facilitating broader epidemiological mapping of EBV patterns across Latin America and distinguishing between regional variations and technical artifacts through future studies employing standardized methodologies across populations.

## 5. Conclusions

This study represents the first comprehensive molecular and immunohistochemical characterization of EBV in lymphoproliferative disorders from Western Mexico, establishing distinct epidemiological patterns that align with Latin American regional characteristics. Key epidemiological findings demonstrate significant associations: neoplastic disorders show substantially higher EBV detection than reactive conditions (53.9% vs. 24.2%), with adults (31–60 years) demonstrating peak detection frequency. The elevated rates observed (80.6% Hodgkin lymphoma; 39.3% non-Hodgkin lymphoma) underscore the critical importance of including Latin American populations in international research initiatives and supporting region-specific diagnostic approaches.

Our combined molecular-immunohistochemical methodology proves feasible in resource-limited settings, demonstrating applicability across similar healthcare environments in developing regions. Future directions include expansion to comprehensive national epidemiological mapping, development of quantitative viral assessment methodologies, and establishment of longitudinal cohorts. This epidemiological foundation represents an essential step toward evidence-based diagnostic strategies and therapeutic applications tailored to Latin American population characteristics, emphasizing the critical need for prospective validation of clinical applications.

## Figures and Tables

**Table 1:** Demographic and clinical characteristics of the study population.

Characteristic	*n* (%)
Sex	
Male	84 (42.0%)
Female	116 (58.0%)
Age Group	
Children (0–15 years)	61 (30.5%)
Young adults (16–30 years)	59 (29.5%)
Adults (31–60 years)	55 (27.5%)
Elderly (>60 years)	25 (12.5%)
LPD Type	
Reactive	124 (62.0%)
Neoplastic	76 (38.0%)
Most Frequent Location	
Cervical	124 (62.0%)
Unspecified	27 (13.5%)
Inguinal	21 (10.5%)
Diagnostic Group	
Lymphoid hyperplasia	91 (45.5%)
Infectious association	33 (16.5%)
Hodgkin lymphoma	31 (15.5%)
Non-Hodgkin lymphoma	28 (14.0%)
Other neoplasms	17 (8.5%)

Mean age: 28.27 years (SD: 22.117); Range: 1–89 years.

**Table 2:** Associations between clinical characteristics and EBV positivity.

Characteristic	EBV(+) *n* (%)	EBV(−) *n* (%)	*p*-Value	OR (95% CI)
Age Group			0.025	
Children	21 (34.4%)	40 (65.6%)		1.00 (Reference)
Young adults	13 (22.0%)	46 (78.0%)	0.832	0.88 (0.36–1.99)
Adults	27 (49.1%)	28 (50.9%)	0.132	1.83 (0.89–3.88)
Elderly	10 (40.0%)	15 (60.0%)	0.630	1.27 (0.50–3.25)
LPD Type			<0.001	
Reactive	30 (24.2%)	94 (75.8%)		1.00 (Reference)
Neoplastic	41 (53.9%)	35 (46.1%)	<0.001	3.67 (1.98–6.69)
Diagnostic Group			<0.001	
Lymphoid hyperplasia	17 (18.7%)	74 (81.3%)		1.00 (Reference)
Infectious association	13 (39.4%)	20 (60.6%)	0.030	2.82 (1.20–6.99)
Hodgkin lymphoma	25 (80.6%)	6 (19.4%)	<0.001	18.14 (6.24–51.13)
Non-Hodgkin lymphoma	11 (39.3%)	17 (60.7%)	0.039	2.81 (1.09–6.798)
Other neoplasms	5 (29.4%)	12 (70.6%)	0.332	1.81 (0.62–5.62)
HL vs. NHL			0.001	6.43 (2.08–19.41)

OR: Odds Ratio; CI: Confidence Interval; LPD: Lymphoproliferative Disorder; HL: Hodgkin Lymphoma; NHL: Non-Hodgkin Lymphoma. Statistical significance was set at *p* < 0.05.

**Table 3:** Distribution of EBV positivity by histological subtypes and diagnostic groups.

Diagnostic Group/Subtype	Total	EBV(+) *n* (%)	EBV(−) *n* (%)	*p*-Value
Hodgkin Lymphoma	31	25 (80.6)	6 (19.4)	0.047
cHL, Mixed Cellularity	10	9 (90.0)	1 (10.0)	
cHL, Nodular Sclerosis	15	12 (80.0)	3 (20.0)	
cHL Lymphocyte Depletion	4	2 (50.0)	2 (50.0)	
cHL, Lymphocyte Rich	2	2 (100.0)	0 (0.0)	
Non-Hodgkin Lymphoma	28	11 (39.3)	17 (60.7)	0.982
DLBCL	10	4 (40.0)	6 (60.0)	
Follicular Lymphoma	5	2 (40.0)	3 (60.0)	
T-cell Lymphomas	4	2 (50.0)	2 (50.0)	
Burkitt Lymphoma	3	1 (33.3)	2 (66.7)	
Other NHL types *	6	2 (33.3)	4 (66.7)	
Infectious Association	33	13 (39.4)	20 (60.6)	0.039
TB-associated lymphadenitis	19	5 (26.3)	14 (73.7)	
Granulomatous lymphadenitis	4	3 (75.0)	1 (25.0)	
Castleman Disease	3	1 (33.3)	2 (66.7)	
Histoplasmosis	2	2 (100.0)	0 (0.0)	
Other infectious conditions †	5	2 (40.0)	3 (60.0)	
Lymphoid Hyperplasia	91	17 (18.7)	74 (81.3)	0.587
Follicular hyperplasia	33	8 (24.2)	25 (75.8)	
Mixed hyperplasia	27	4 (14.8)	23 (85.2)	
Other reactive patterns ‡	31	5 (16.1)	26 (83.9)	

* Includes mantle cell, lymphoblastic, plasmacytoid, and high-grade NOS lymphomas. † Includes MAC infection, BCGitis, and necrotizing lymphadenitis. ‡ Includes sinusal hyperplasia, dermatopathic changes, and other non-specific patterns. cHL = classical Hodgkin lymphoma; NHL = non-Hodgkin lymphoma; DLBCL = diffuse large B-cell lymphoma; TB = tuberculosis. Statistical significance was set at *p* < 0.05.

**Table 4:** Diagnostic agreement between PCR-BNTp143 and LMP-1 immunohistochemistry.

Diagnostic Group	Total	LMP-1-Positive	PCR-Positive	EBV(+) Overall
Hodgkin Lymphoma	31	15 (48.4%)	19 (61.3%)	25 (80.6%)
Non-Hodgkin Lymphoma	28	5 (17.9%)	8 (28.6%)	11 (39.3%)
Other Neoplasms	17	1 (5.8%)	4 (23.5%)	5 (29.5%)
Infectious Association	33	0 (0.0%)	13 (39.4%)	13 (39.4%)
Lymphoid Hyperplasia	91	2 (2.2%)	17 (18.7%)	17 (18.7%)
Total	200	23 (11.5%)	61 (30.5%)	71 (35.5%)

Polymerase chain reaction (PCR) assays detect the presence of viral genomic material, offering high sensitivity even in latent infections or in complex microenvironments where viral load may be low. In contrast, immunohistochemistry (IHC) identifies viral protein expression, serving as a direct marker of active infection within specific cell populations. Thus, while PCR can reveal viral DNA independently of transcriptional activity, IHC provides functional evidence of viral protein synthesis, indicating viral status.

**Table 6:** International comparison of EBV detection rates in lymphoproliferative disorders by geographical region and methodology.

Region/Country	Study	Year	Lymphoma Type	Detection Rate (%)	Method	Population Size
Brazil	[5]	2018	HL	65–75	EBER-ISH	324
China	[7]	2013	HL	74.6	PCR	59
				67.8	EBER-ISH	
Ethiopia	[20]	2023	HL	99.0	qPCR	91
Rwanda	[21]	2022	HL	54	EBER-ISH	52
Zambia	[22]	2018	HL	40.9	PCR	22
Mexico (Central)	[23]	2013	HL	71.4	EBER-ISH	42
Peru	[24]	1993	HL	90.0	EBER-ISH	20
France	[25]	2022	HL	31.0	EBER-ISH	1248
United States	[26]	1997	HL	36.0	EBER-ISH	154
Germany	[27]	1991	HL	42.0	EBER-ISH	95
Ethiopia	[20]	2023	NHL	99.0	qPCR	197
Rwanda	[21]	2022	NHL	9	EBER-ISH	207
Zambia	[22]	2018	NHL	54.5	PCR	98
Mexico (South-Central)	[19]	2011	DLBCL	7.0	EBER-ISH	43
Germany	[19]	2011	DLBCL	8.0	EBER-ISH	50
France	[25]	2022	DLBCL	5.0	EBER-ISH	2156
Brazil	[28]	2018	BL	92.0	EBER-ISH	Multiple
Argentina	[28]	2018	BL	83.0	EBER-ISH	Multiple
Ghana	[28]	2018	BL	94.0	EBER-ISH	Multiple
Korea	[29]	2020	ENKTL	100.0	EBER-ISH	287
China	[30]	2014	ENKTL	98.5	EBER-ISH	132

EBV detection rates across different geographical regions and lymphoma subtypes. Data compiled from studies using standardized detection methods. HL: Hodgkin Lymphoma; NHL: Non-Hodgkin Lymphoma; DLBCL: Diffuse Large B-Cell Lymphoma; BL: Burkitt Lymphoma; ENKTL: Extranodal NK/T-Cell Lymphoma; EBER-ISH: Epstein–Barr Virus-Encoded Small RNAs In Situ Hybridization; PCR: Polymerase Chain Reaction; qPCR: Quantitative Real-Time PCR.

## Data Availability

The data presented in this study are available on request from the corresponding author. The data are not publicly available due to privacy restrictions and institutional policies regarding patient information.

## Abbreviations

The following abbreviations are used in this manuscript:

EBV	Epstein–Barr Virus
EBER-ISH	Epstein–Barr Virus-Encoded Small RNAs In Situ Hybridization
LMP-1	Latent Membrane Protein 1
EBNA2	Epstein–Barr Nuclear Antigen 2
PCR	Polymerase Chain Reaction
qPCR	Quantitative Real-Time PCR
RT-PCR	Reverse Transcription PCR
GAPDH	Glyceraldehyde 3-phosphate dehydrogenase
LPD	Lymphoproliferative Disorder
HL	Hodgkin Lymphoma
NHL	Non-Hodgkin Lymphoma
cHL	Classical Hodgkin lymphoma
DLBCL	Diffuse Large B-Cell Lymphoma
ENKTL	Extranodal NK/T-Cell Lymphoma
BL	Burkitt Lymphoma
FFPE	Formalin-Fixed Paraffin-Embedded
IHC	Immunohistochemistry
HE	Hematoxylin and Eosin
